# Waka ama: An exemplar of indigenous health promotion in Aotearoa New Zealand

**DOI:** 10.1002/hpja.632

**Published:** 2022-06-28

**Authors:** Angelique Reweti, Christina Severinsen

**Affiliations:** ^1^ Massey University Palmerston North New Zealand

**Keywords:** community development, health promotion, health/wellbeing, indigenous, Māori

## Abstract

**Issue addressed:**

The use of old‐style, top‐down health education and awareness programmes in Aotearoa New Zealand, which adopt a single issue‐based approach to health promotion, primarily ignores a broad approach to social determinants of health, as well as indigenous Māori understandings of wellbeing.

**Methods:**

This paper draws on the indigenous framework Te Pae Māhutonga as a guide for presenting narratives collated from members of a waka ama rōpū (group) who were interviewed about the social, cultural and health benefits of waka ama.

**Results:**

This waka ama case study is an exemplar of community‐led health promotion within an indigenous context, where Māori values and practices, such as whanaungatanga (the process of forming and maintaining relationships), manaakitanga (generosity and caring for others) and kaitiakitanga (guardianship), are foundational. The findings highlight the multiple benefits of engagement in waka ama and illustrate effective techniques for enhancing wellbeing within local communities.

**Conclusion:**

At a time when Aotearoa New Zealand is seeing a decreasing trend in physical activity levels and an increase in mental health challenges, waka ama provides us with an exemplar of ways to increase health and wellbeing within our communities.

**So what?:**

The findings of this research contribute to the evidence base of effective indigenous health promotion, bridging the gap between academia and local community action. To better recognise, comprehend and improve indigenous health and wellbeing, we argue that active participation of people in the community is required to achieve long‐term and revolutionary change.

## 1 INTRODUCTION

Health promotion includes a wide variety of actions performed in diverse contexts. Rather than focusing on individual lifestyle health promotion, the New Public Health movement emphasises community involvement and structural and environmental change as the primary means of improving health.^1^ However, it is argued that there has been some drift away from these original New Public Health broad and holistic understandings of health promotion over time. Baum and Fisher^2^ argue that health promotion remains narrowly and unduly focused on individual behaviour change strategies in spite of evidence that health is determined by wider social determinants.

As is the case with other indigenous people worldwide, Māori are disproportionately affected by lifestyle‐related illnesses and continue to experience persistent health and social inequities when compared to others living in Aotearoa New Zealand.^3^, ^4^ Disparities in avoidable diseases between Māori and Pākehā (non‐indigenous population of Aotearoa New Zealand) are significantly linked to broader socio‐cultural/political determinants of health, such as income, housing and education. These broader variables often underpin specific behaviours that we attribute to be causative of chronic illness, such as increased alcohol, sugar, stress and inactivity. As a result of these discrepancies, current discourses hold indigenous peoples responsible for their own ill health.^3^ The continuous use of traditional, top‐down health education and awareness campaigns in Aotearoa New Zealand primarily ignores a broad approach to social determinants of health, as well as indigenous Māori concepts of wellbeing.^3^

Indigenous Māori understandings of health are holistic, take a collectivist approach and use cultural beliefs and values to guide action.^5^, ^6^ Māori socioecological approaches, such as Te Whare Tapa Whā,^7^ Te Wheke^8^ and Te Pae Māhutonga^9^, ^10^ consider the social and physical environment as well as participation within settings. Instead of focusing on those at risk for a specific disease, an indigenous perspective considers individuals and their whānau (extended family) within their social and everyday circumstances. In contrast to individual health promotion, which is dominated by an emphasis on individual lifestyle choices, community and settings‐based health promotion incorporates participatory community‐level interventions. This type of approach respects that people and communities have the right to define what health means for them and are empowered to have control over decision‐making processes which impact their health.^5^

This qualitative case study presents the experiences of paddlers from a local waka ama rōpū (outrigger canoe club) in Heretaunga, Aotearoa New Zealand. Grounded in mātauranga (Māori bodies of knowledge), waka ama (outrigger canoe) is an increasingly popular sport in Aotearoa New Zealand.^11^ Established in 2001, Heretaunga Ararau o Ngāti Kahungunu Waka Ama Rōpū (name of the waka ama club) has paddlers of all ages, including midgets (5 years of age) through to golden masters (70 years and older). Alongside weekly paddling sessions and opportunities to participate in local, national and international waka ama competitions, paddlers are involved in the organisation and running of the rōpū and conservation activities in the environments in which they operate. Beyond the physical health benefits for paddlers, waka ama enhances wellbeing, connecting paddlers to each other through whanaungatanga (process of forming and maintaining relationships) and manaakitanga (generosity and caring for others), and to the environment through the practice of kaitiakitanga (guardianship).

To illustrate the benefits of waka ama as an exemplar of indigenous health promotion, we use Te Pae Māhutonga,^10^ an indigenous health promotion model developed by Emeritus Professor Sir Mason Durie based on the constellation of stars commonly referred to as the Southern Cross. Visible low in the night sky and identifying the magnetic south pole, Te Pae Māhutonga is well‐known as a navigational tool and closely associated with the discovery of Aotearoa New Zealand.^9^, ^10^ Drawing on influences from Maui Pōmare (first Māori medical practitioner in Aotearoa New Zealand) and the Ottawa Charter,^12^ Durie uses the imagery of Te Pae Māhutonga as a way to conceptualise Māori health promotion. The constellation has four central stars arranged in the shape of a cross, and two pointer stars directed towards the cross. The central stars of Te Pae Māhutonga are used to represent four key tasks of health promotion: mauriora (secure cultural identity), waiora (environmental protection), toiora (healthy lifestyles) and te Oranga (participation in society). The two stars pointing toward the cross formation represent the prerequisites for engagement, ngā Manukura (community leadership) and te Mana Whakahaere (autonomy).^9^, ^10^ This research into the social, health and cultural benefits of waka ama reminds us that it is not always top‐down, service and intervention‐focused approaches that our communities' benefit from the most. Rather, it encourages us to think about how we can draw upon the rich resource of mātauranga Māori (bodies of knowledge) and expertise already within our local communities. Māori terms and concepts are used throughout this article. Following each instance of a new Māori word or term, an English translation is provided, as well as a glossary provided in the Appendix.

## 2 METHODS

### 2.1 Philosophical approach and research design

The purpose of this qualitative case study was to explore the social, cultural and health benefits experienced by paddlers involved with a local waka ama rōpū. Research design and interpretation were informed by a kaupapa Māori approach, prioritising a Māori worldview in research design and interpretation. A kaupapa Māori approach seeks to empower communities by validating local knowledge systems and experiences and by ensuring that those involved in the research gain benefits as a result.^13^, ^14^, ^15^, ^16^

### 2.2 Ethics

Ethical consideration for this project was informed by tikanga (Māori principles that inform practice) of the waka ama rōpū and by following guidelines and procedures set out by Massey University Health and Ethics Committee (MUHEC). Cultural safety, informed consent, privacy and confidentiality issues, access to and ownership of data and how data might be used were all considered. The project was evaluated by members of the waka ama rōpū and peer‐reviewed by academic colleagues and was determined to be low risk. The project was recorded in the MUHEC database, Ethics Notification Number: 4000018859.

### 2.3 Participants and recruitment

An established relationship between the primary researcher and Heretaunga Ararau o Ngāti Kahungunu Waka Ama Rōpū led to researchers being invited to attend two training sessions to meet with club members to gain more insight into the club's activities. The waka ama rōpū facilitated the involvement of its members, resulting in 16 club members being interviewed kanohi ki te kanohi (face to face) at the club home base in Clive and at a local café affiliated with the waka ama rōpū. Nine of these members were also involved in short interviews that were filmed for the purpose of developing a short film alongside video footage captured following two‐morning training sessions.^17^ Paddlers ranged in age from 35 to 65 years of age, which included seven women and nine men. Paddlers were of mixed ethnicity and experience levels, with some who were relatively new to waka ama and others who had participated for many years.

### 2.4 Data collection and analysis

The researchers employed a semi‐structured interview approach to facilitate the flow of conversation and allow researchers and paddlers to explore specific themes and responses in greater depth. As a topic guide, participants were asked about their motivations to join waka ama, what waka ama means to them, what changes they have noticed in themselves since starting waka ama and about the social, cultural and health benefits of being involved in waka ama. Participants provided verbal and written consent for the interviews to be recorded. Interviews were transcribed verbatim and thematic analysis was carried out to identify, analyse and report patterns of meaning. Using topic categories relating to health and wellbeing, the transcripts were coded according to the information they contained. In this process, codes of similar nature were grouped to create concepts, and from these concepts, key themes were evaluated and further refined according to feedback from the waka ama rōpū. As part of the process of identifying key themes, it was noted that they mapped well to Te Pae Māhutonga model for health promotion, and therefore we chose to structure narratives based on this framework.

## 3 RESULTS

Based on Te Pae Māhutonga, results are presented under the four key components of health promotion, mauriora (access to te ao Māori), waiora (environmental protection), toiora (healthy lifestyle) and te oranga (participation in society) and the two pointer stars representing ngā manukura (leadership) and mana whakahaere (autonomy). A summary of the results is presented in Table 1. Benefits of waka ama.

**TABLE 1:** Benefits of waka ama

Mauriora: Access to te ao Māori	Positive integration and normalisation of te ao Māori in experiential setting Secures cultural identity for both Māori and non‐Māori
Waiora: Environmental protection	Increased environmental awareness and engagement in environmental protection activities Affirms identity and place of belonging in relation to the environment Understanding the relationship between environmental and human health and wellbeing
Toiora: Healthy lifestyles	Integrates healthy choices into daily activities Promotes smoke‐free, alcohol‐free and sugar sweetened beverage free environments Intergenerational activity enjoyed across age groups
Te Oranga: Participation in society	Builds social cohesion across diverse groups of people Broadens community networks and engagement
Ngā manukura: Leadership	Leadership fostered from within the flax roots Enables leaders to situate themselves in the context of the communities that they serve
Mana whakahaere: Autonomy	Strengthens the capacity of local people within the context of their communities Ability to realise aspirations through active roles in governance, organisation, decision‐making, kaitiakitanga and growth of rōpū

### 3.1 Mauriora: Access to te ao Māori

Mauriora refers to access to te ao Māori (Māori world) recognising the critical role of cultural identity in the health and wellbeing of a people. Te Ao Māori is holistic and cyclical, with everything connected to everything else through whakapapa. Within this worldview, the waka is positioned as a living component of Māori people, the land and water shared and the history and traditions that each generation carries forwards. Paddlers learn and use karakia (incantations), waiata (song) and te reo (Māori language) as part of their everyday practice on the water providing a pathway for many to learn and engage in Te Ao Māori.^18^ Paddlers demonstrate an understanding around waka as having their own mauri (physical vitality) and as such learn about the appropriate language and protocols to follow to respect the waka, the environment and their fellow paddlers. These efforts have led to an increased number of members in the rōpū becoming aware of the importance of tikanga with many going on to expand their knowledge of te reo and mātauranga Māori through courses at the local wānanga (tertiary institute).

*(It's) a way into tikanga, which is not sort of academic or heady. It's just about participation and about doing things (Male paddler)*.

*We always have a karakia before we go for a paddle. And just through like respecting the waka as entities. It's not just a boat, you know. It's an entity. It has a gender and requires due respect to be shown to it. You know, the tikanga around what that is, so that opens the door to understanding that things are not just objects (Female paddler)*.

Waka ama also helps to create a safe space for experiential learning and partnership between Māori and non‐Māori providing an opportunity for Pākehā New Zealanders to discover and enhance their own sense of identity relative to Māori culture. Pākehā paddlers discussed how waka ama provides a gateway into te ao Māori that normalises Māori culture demonstrating ways te ao Māori can be incorporated into their daily activities.

*It's been a learning curve for me. To the extent that I'm hoping to learn te reo [Māori]. And just the whole thing. It's brought me closer to Māoridom and opened my eyes to that side of it. That's been great, yeah. I've really enjoyed it (Male paddler)*.

*As a Pākehā, there's not many in roads into Māori culture, unless you are born into it, or married into, or quite strong‐willed to get involved yourself… so through waka ama, there is, and I've gone on to do the course through the wānanga, which is great for learning karakia and waiata, and being away on noho marae and stuff like that, which I would not have experienced through any other way… so I think that it's a real positive thing for non‐Māori (Male paddler)*.

### 3.2 Waiora (environmental protection)

Waiora reflects how our health and wellbeing as a people are connected and influenced by our interactions with the external environment. Waka ama provides opportunities for paddlers to become immersed in the natural environment enabling paddlers to make both physical and spiritual connections. Paddlers were open and expressive about how they felt the connection with the environment impacted their health and wellbeing.

*You're outside. You're connecting with the awa and the moana. And just connecting with nature, so that has a really positive effect on your mental health. (Male paddler)*.

*To be on the water, to be out in the elements. Late in the evening, early in the morning, you know, it's absolutely magical (Male paddler)*.

Natural features such as awa (rivers) and maunga (mountains) form important aspects of Māori identity, often used to define tribal boundaries and commonly recalled during pepeha (a Māori introduction). Engaging with the awa has helped paddlers to affirm their identity and place of belonging in their local community.

*It's really helped me… we have lived here for quite a few years, but somehow being on the water and being connected to the river, seeing it in that way, it's really helped me be in a relationship with this land and place more deeply. (Female paddler)*.

As a task for health promotion, waiora places a strong emphasis on environmental protection. It is about finding mutually beneficial ways to engage with the environment which maintain and preserve natural integrity and resources for the benefit of ensuing generations. Waka ama helps fosters a reciprocal relationship between paddlers and the environment, encouraging individuals to recognise their role as kaitiaki (guardians) in caring for the environment that nourishes them.

*A lot of people that are into waka ama are also very much into protecting these waterways or fixing them or getting change to happen, because they are in direct contact with them… and to me that's a real Māori thing, you know everything is connected to the land, connected to the water, and so that part I really value as well. (Male paddler)*.

### 3.3 Toiora (healthy lifestyles)

Toiora is about facilitating healthy lifestyles. Within a te ao Māori perspective, maintaining and fostering healthy lifestyles is about enhancing spiritual, mental, physical and collective health, and as discussed under Section 3.2, indigenous health promotion also recognises engagement in the environment as influencing health and wellbeing. While there are obvious physical benefits to participating in waka ama, there are also other benefits, such as a noticeable increase in individuals' self‐confidence, with correlations being formed between improved physical ability and improved mental wellbeing.

*I wasn't very fit when I started, and now I feel like I'm fitter and stronger than I've been all my life really. I've got way more energy, way more stamina. And you know it gives you a good head space (Female paddler)*.

*It has a really positive effect on your mental health (Male paddler)*.

*I'm fitter now and stronger now in my 50s than I have been for 20 years. So it's kind of given me a lot more confidence to trust my body whereas I used to start to worry about whether it was going to break on me (Male paddler)*.

Paddlers also spoke of the importance of having a positive, strength‐based approach to wellbeing within the rōpū. They hold collective aspirations on their journeys to become healthier together. This allows them to realise their aspirations as a group. For many members, it has given them a focus and provided opportunities to realise their potential by competing together at regional, national and international levels. In a whānau atmosphere, their participation has fostered a strong sense of kotahitanga (unity) and manaakitanga (generosity and caring for others), teaching participants a sense of collective responsibility and commitment towards their rōpū:

*Being able to train as a team together, has been fantastic, because everybody just motivates each other. So health‐wise, it's been great (Female paddler)*.

*It's a team sport. You either all do it, and you all get better or you do not. And if one person does not get better, then that's the responsibility of everybody (Male paddler)*.

Waka ama also encourages intergenerational participation with a wide range of age groups being involved in the initiative.

*It's very family oriented. You know, you can have three generations all paddling, which is awesome. It's a very inclusive environment (Female paddler)*.

Another way that waka ama encourages healthy lifestyles is by establishing smoke‐free, alcohol‐free and sugar‐sweetened beverage‐free zones at all waka ama National events.

*I've done a whole bunch of other sports and, you know, too much drinking and smoking and other rubbish goes with it. But there's nothing like that in this sport (Male paddler)*.

*The healing thing I've found about waka ama events is that you go to the sprint nationals in Karāpiro, and it's a huge event, I think they had 3000 odd paddlers last time, from all ages, but the thing is, all these food outlets, there's no fizzy, there's no smoking, there's no junk food, it's all a real push for those healthy lifestyle food choices and it just becomes the norm you know (Male paddler)*.

### 3.4 Te Oranga (participation in society)

Te Oranga is about fostering inclusion and participation in society. It is about making space for community voices to be heard and acted upon and ensuring health promotion activities facilitate participation in wider society. Waka ama enables and encourages members to become involved in their community. As an intergenerational activity, it incorporates all age groups and brings together people from diverse backgrounds. Through a number of free waka ama activities, Heretaunga Ararau o Ngāti Kahungunu is instrumental in growing health and awareness within their community.

*I went to an event last night where there were 80 people who were trying waka for the first time. So there was a huge community of people coming. And I just think that waka is a community‐building activity (Male paddler)*.

*Great people to paddle with. And they are people that I probably would not necessarily cross paths with otherwise, and so your life is much richer for that (Female paddler)*.

For some paddlers, waka ama has acted as an intermediary enabling community relationships to be instigated and strengthened based on mutual experiences of waka ama. For example, a school teacher noticed improved engagement with her students after sharing mutual experiences of waka ama while a medical professional found her experience in waka ama has broadened her community networks enabling a more consolidated approach to engaging with the people in the community she works in.

*Paddling waka ama has been a really great thing that I can talk about with the people that come into my practice, because it makes them give me a second chance in a way. Give me a second look as the nurse, as another way of connecting with the people that I'm working with in a positive, healthy way and coming into more real and deeper connection and understanding, not just at a superficial level, it's been quite big for me in that way (Female paddler)*.

### 3.5 Ngā manukura: Leadership and Mana whakahaere: Autonomy

Directing the gaze towards the four health promotion goals are the two pointer stars represented as ngā manukura (leadership) and mana whakahaere (autonomy). These attributes are seen as prerequisites providing guidance on how we might move toward aspirations within Te Pae Māhutonga. Ngā manukura (leadership) from a te ao Māori perspective is about elevating and serving the needs of others.^9^, ^10^, ^19^ Mana whakahaere refers to a community's ability to exercise autonomy and self‐determination in promoting its own health.^9^, ^10^, ^20^ Aspirations should arise from within the community with actions consistent and responsive to local and cultural contexts.^10^

The collective, strength‐based nature of waka ama builds the organisational capacity of local people within the context of their communities. Paddlers engage not just in the physical activity of paddling, but play roles in governance, organisation, decision‐making, kaitiakitanga, learning, teaching and education, leading and sharing. Waka ama is sustained from the flax roots (at a local level) with many volunteers supporting the rōpū throughout the weekly club activities and participation in national and international events. Paddlers spoke of the vast opportunities they experienced through waka to grow and develop, not only their personal skills but opportunities for the club to grow and expand its outreach within the community.

*It's not just the paddling, we are involved in a lot of other things, projects through the club, so that's part of it (Male paddler)*.

*It's really challenged me in lots of ways, all the different relationships, different types of people, being on the committee, so it's brought a lot of growth in lots of ways (Female paddler)*.

*Everybody is encouraged to share their knowledge and their experiences. And they do, they are all imparting knowledge, and for that, we are all very grateful. That's how the club grows (Female paddler)*.

## 4 DISCUSSION

Results demonstrate how waka ama aligns to Te Pae Māhutonga, validating an indigenous Māori understanding of wellbeing in a number of ways. Aligned to the principle of mauriora, findings show that waka ama is an example of a health‐promoting practice that contributes to securing cultural identity and overall wellbeing. Participants are provided with a gateway into te ao Māori and a safe space to learn about and explore their cultural identity in relation to the teachings of tikanga Māori. Studies have shown that cultural practices have an inextricable link to health and wellbeing, with evidence that a secure cultural identity enhances mental wellbeing and alleviates symptoms of depression.^21^, ^22^, ^23^, ^24^, ^25^ This shows that securing cultural identity is an important determinant of health from an indigenous perspective. Activities that facilitate meaningful and positive experiences with culture, such as waka ama, can contribute to an individual's sense of cultural identity and wellbeing.

Water activities like waka ama support the principle of waiora by providing opportunities for participants to immerse themselves in the environment. Understanding the interdependence of environmental and human health is a core concept in te ao Māori and research has shown that engaging with nature is beneficial to overall health and wellbeing.^10^, ^26^, ^27^ This interdependence relates to the idea that the mauri of the environment has an effect on the mauri of the people and vice versa.^8^, ^26^ There are a number of variables that have a negative impact on health and wellbeing, including pollution, depletion of natural resources and the loss of land.^28^ Living in cities can also promote detachment and a lack of appreciation for the natural environment. Participation in activities like waka ama helps connect people with their natural surroundings, which can lead to positive changes in how they interact and view their relationships with the environment. Having direct contact with water increases environmental awareness and motivation for waka ama members to participate in environmental protection activities, ultimately leading to improved health and wellbeing for both people and the environment.

Waka ama is a healthy, fun, intergenerational activity incorporating a number of healthy lifestyle benefits that link both physical and spiritual dimensions of wellbeing. This is consistent with the principle of toiora, which emphasises opportunities to promote healthy lifestyles.^9^, ^10^ Benefits of intergenerational programmes and their ability as being effective in supporting change in health behaviours have been widely supported by research.^29^, ^30^, ^31^ In Aotearoa New Zealand, mainstream healthcare services are primarily focused on the individual and are more concerned with the physical elements of health than with any other aspect.^4^ Waka ama offers an alternative to these types of individualised programmes by emphasising the value of a whānau‐like environment and the myriad other dimensions of health, such as emotional and spiritual wellbeing, that contribute to overall wellbeing. Research demonstrates that programmes focused on cultural concepts of whānau and enhancing the collective good resonate more strongly with Māori than programmes emphasising individual accomplishment.^3^, ^32^ Participation in waka ama supports these findings, with fundamental Māori concepts such as whanaungatanga, manaakitanga, kotahitanga and kaitiakitanga underpinning waka ama's success as a sustainable health promotion initiative.

The culture of waka ama also encourages smoke‐free, alcohol‐free and sugar‐sweetened beverage‐free environments. Despite the fact that unhealthy food and beverage marketing has been identified as a major contributor to obesity and adverse health conditions, sports sponsorship by the fast‐food industry remains a widespread practice and represents millions of dollars in advertising expenditures.^33^ For example, alcohol promotion at sporting events and engagement of clubs and players in alcohol‐related activities such as drinking games and alcohol prizes have all been linked to increased alcohol consumption.^34^, ^35^ Restricting alcohol promotion, including sports sponsorship, is a highly successful and cost‐efficient method for reducing alcohol harm.^36^ In a society where many indigenous peoples engage in risk‐laden lifestyles such as drinking, smoking and gambling, positive role modelling, such as that exemplified by the kaupapa of waka ama, can serve to promote healthier lifestyles.

As a community activity, waka ama facilitates social cohesion across diverse social groups which are in accordance with the principle of te oranga. Participants demonstrated a strong commitment to the rōpū beyond just going out on the water to paddle which broadens individual's community networks and engagement. This is especially important for Māori, who regularly experience discrimination from different levels of society, including in health, education and politics.^37^, ^38^ For example, there is increasing evidence that Māori and non‐Māori differ in terms of access to both primary and secondary health care services.^39^, ^40^ Often non‐Māori staff bring attitudes and perceptions to health care delivery that reflect little understanding of Māori realities and cultural values.^41^ The establishment of relationships based on a similar kaupapa, such as waka ama, can help break down implicit bias barriers for both health providers and patients, signifying the crucial role initiatives like waka ama generate in their local communities.

The principles of ngā manukura and mana whakahaere are promoted in waka ama by providing paddlers with opportunities to engage in various leadership and governance roles as well as by creating an environment that facilitates the development of potential within the rōpū. Leadership fostered from within the flax roots enables leaders to situate themselves in the context of the communities that they serve.^19^ This is consistent with the requirement of mana whakahaere that ownership of activities and growth objectives be firmly rooted in the communities in which health promoters work. For example, public agencies and health practitioners have frequently assumed leadership roles on behalf of Māori, which is a common indigenous experience.^10^ To promote the growth and sustainability of health initiatives, we argue that health practitioners need to shift their focus to organic, community‐based health promotion that already exists within communities and to rely on flax root leadership, such as that developed through waka ama.

These findings contribute to the evidence base of effective indigenous health promotion, bridging the gap between academia and local community action. At a time when Aotearoa New Zealand is seeing a decreasing trend in physical activity levels and an increase in mental health challenges,^3^, ^42^ waka ama provides us with an exemplar around positive ways to increase health and wellbeing within our communities. This initiative is in contrast to individual behaviour‐driven health promotion activities that focus on education and awareness, and top‐down single‐issue interventions directed at communities. While there is a shift towards a more holistic approach to health promotion in Aotearoa New Zealand, current funding models which determine outcome measures rarely reflect anything other than biophysical aspects of health.^3^ Te Pae Māhutonga incorporates a holistic approach to health promotion focusing on a range of health determinants grounded in indigenous knowledge. It teaches us to look beyond the individual and particular markers of illness and symptoms and to consider how we might incorporate Indigenous knowledge and values in a contemporary health promotion setting.^3^, ^10^

## 5 CONCLUSION

Using Te Pae Māhutonga as a guide, the findings of this research highlight the multiple benefits of engagement in waka ama and illustrate effective techniques for enhancing wellbeing within local communities. Positioned within a Māori worldview, waka ama enhances physical fitness while at the same time promoting cultural identity, social connectedness, intergenerational participation and community cohesion. It stands in stark contrast to many health promotion programmes, which continue to be narrowly focused on individual behaviour modification as their primary objective. The research shows how indigenous health promotion activities like waka ama can help people adopt healthier lifestyles collectively in a sustained manner. This shift in focus from individual behaviour change strategies toward community‐based health promotion initiatives is critical to the long‐term viability of these programmes since it is through this process of community participation that culturally relevant and meaningful programmes are created. In addition, we believe that long‐term success requires flax root leadership, such as that generated through waka ama.

This may necessitate some health practitioners to abandon established notions of authority to consider strategies to best support the intrinsic health potential of their communities. This requires a strong commitment to building relationships and working with and on behalf of the communities in which health practitioners are involved, and considering the promotion of health holistically instead of focusing on narrowly defined issue‐based health promotion. In addition, this approach has implications for funding models, which tend to favour the implementation of individual behaviour change strategies on a national scale over developing capacities at a localised community level. Improving responsiveness to local priorities will require a better understanding of how funding mechanisms can better support community‐based health promotion. Overall, to better recognise, comprehend and improve indigenous health and wellbeing, this article advocates for indigenous knowledge to be more fully integrated within health promotion which can be achieved through using frameworks such as Te Pae Māhutonga.

## CONFLICT OF INTEREST

The authors declare no conflict of interest.

## GLOSSARY OF MĀORI TERMS

Te reo (Māori language) does not always readily translate into English words, therefore, this glossary is not definitive and refers to the context of te reo used in this publication only.

**Table:**

Awa	River
Heretaunga Ararau o Ngāti Kahungunu Waka Ama Rōpū	name of the waka ama club
kaitiakitanga	guardianship
karakia	incantations
kanohi ki te kanohi	face to face
kotahitanga	unity
manaakitanga	generosity and caring for others
Māori	Indigenous to Aotearoa New Zealand
mātauranga	Māori bodies of knowledge
maunga	mountain
mauri	physical vitality
Pākehā	non‐indigenous population of Aotearoa New Zealand
pepeha	a Māori introduction
rōpū	group
te ao Māori	Māori world
tikanga	Māori principles that inform practice
waiata	song
waka ama	outrigger canoe
whānau	extended family
whanaungatanga	process of forming and maintaining relationships
